# Benign Paroxysmal Positional Vertigo after Dental Procedures: A Population-Based Case-Control Study

**DOI:** 10.1371/journal.pone.0153092

**Published:** 2016-04-04

**Authors:** Tzu-Pu Chang, Yueh-Wen Lin, Pi-Yu Sung, Hsun-Yang Chuang, Hsien-Yang Chung, Wen-Ling Liao

**Affiliations:** 1 Department of Neurology/Neuro-Medical Scientific Center, Taichung Tzu Chi Hospital, Buddhist Tzu Chi Medical Foundation, Taichung, Taiwan; 2 School of Medicine, Tzu Chi University, Hualien, Taiwan; 3 Department of Dentistry, Taipei Medical University Hospital, Taipei, Taiwan; 4 Department of Physical Medicine and Rehabilitation, Taichung Tzu Chi Hospital, Buddhist Tzu Chi Medical Foundation, Taichung, Taiwan; 5 Department of research, Taichung Tzu Chi Hospital, Buddhist Tzu Chi Medical Foundation, Taichung, Taiwan; 6 Department of Dentistry, Taichung Tzu Chi Hospital, Buddhist Tzu Chi Medical Foundation, Taichung, Taiwan; Navodaya Dental College and Hospital, mantralayam Road, INDIA

## Abstract

**Background:**

Benign paroxysmal positional vertigo (BPPV), the most common type of vertigo in the general population, is thought to be caused by dislodgement of otoliths from otolithic organs into the semicircular canals. In most cases, however, the cause behind the otolith dislodgement is unknown. Dental procedures, one of the most common medical treatments, are considered to be a possible cause of BPPV, although this has yet to be proven. This study is the first nationwide population-based case-control study conducted to investigate the correlation between BPPV and dental manipulation.

**Methods:**

Patients diagnosed with BPPV between January 1, 2007 and December 31, 2012 were recruited from the National Health Insurance Research Database in Taiwan. We further identified those who had undergone dental procedures within 1 month and within 3 months before the first diagnosis date of BPPV. We also identified the comorbidities of the patients with BPPV, including head trauma, osteoporosis, migraine, hypertension, diabetes, hyperlipidemia and stroke. These variables were then compared to those in age- and gender-matched controls.

**Results:**

In total, 768 patients with BPPV and 1536 age- and gender-matched controls were recruited. In the BPPV group, 9.2% of the patients had undergone dental procedures within 1 month before the diagnosis of BPPV. In contrast, only 5.5% of the controls had undergone dental treatment within 1 month before the date at which they were identified (*P* = 0.001). After adjustments for demographic factors and comorbidities, recent exposure to dental procedures was positively associated with BPPV (adjusted odds ratio 1.77; 95% confidence interval 1.27–2.47). This association was still significant if we expanded the time period from 1 month to 3 months (adjusted odds ratio 1.77; 95% confidence interval 1.39–2.26).

**Conclusions:**

Our results demonstrated a correlation between dental procedures and BPPV. The specialists who treat patients with BPPV should consider dental procedures to be a risk factor, and dentists should recognize BPPV as a possible complication of dental treatment.

## Introduction

Benign paroxysmal positional vertigo (BPPV) is the most common form of vertigo in the general population with a lifetime prevalence of 2.4%[1]. It is thought to be caused by dislodgement of otoliths from otolithic organs into the semicircular canals. BPPV can be directly induced by head trauma[2] and other inner ear disorders such as vestibular neuritis[3]. However, most etiologies of BPPV are unclear. The potential risk factors for idiopathic BPPV include old age, bed rest[4], migraine[5], osteoporosis[6], and vascular risk factors[1]. In addition, iatrogenic BPPV induced by specific types of surgery[7, 8] has also been observed.

Dental procedures are considered to be a possible cause of BPPV, and even the most common iatrogenic cause[9]. However, previous studies on the correlation between BPPV and dental work have been mostly case reports or case series[10–14], and this study is the first nationwide population-based case-control study conducted to investigate the correlation between BPPV and dental procedures.

## Materials and Methods

### Database

This population-based case-control study used data from the National Health Insurance Research Database (NHIRD) in Taiwan. The NHIRD contains records of approximately 23 million enrollees dating back to March 1995, representing almost 99% of the total population in Taiwan. We reviewed records from the Longitudinal Health Insurance Database (LHID) which includes claims data for 1 million enrollees randomly selected from all beneficiaries of the National Health Insurance program. The medical records included in the LHID include those from as far back as 1996 and are updated annually. In order to ensure confidentiality, the enrollees' personal information is scrambled using anonymous identification numbers. Patient consent is not required to access the NHIRD. This study was approved by the Institutional Review Board of the Buddhist Taichung Tzu Chi General Hospital, Taiwan (REC104-11). We extracted data based on International Classification of Diseases, Ninth Edition, Clinical Modification (ICD-9-CM) codes.

### Study Sample

Patients aged 20 years or older who were diagnosed with BPPV (ICD-9-CM: 386.11) between January 1, 2007 and December 31, 2012 were enrolled. The diagnosis of BPPV was mostly established by board-certified otolaryngologists or neurologists after assessing the medical history of the patients and the results of Dix-Hallpike or supine roll tests. In order to increase the diagnostic accuracy, the patients who were diagnosed with BPPV at least three times in out-patient department follow-up visits or who were hospitalized with BPPV as the primary diagnosis were enrolled as the case group. We excluded the patients who had other vertigo-related diagnoses (ICD-9-CM: 078.81, 386.0–386.10, 386.12–386.9, 780.4) to avoid misdiagnoses of BPPV. The index date was defined as the first diagnosis date of BPPV during the inclusion period for each patient.

We randomly selected individuals without vertigo-related diagnoses (ICD-9-CM: 078.81, 386.0–386.9, 780.4) during the same period from the database as the control group, and matched them with the case patients at a control-to-case ratio of 2:1 according to exact age and gender.

### Study Variables

The patients who had previously received any dental procedure were defined as having undergone a dental procedure, and we then identified those who had undergone the procedure within 1 month and within 3 months before the index date. Dental procedures were further classified into five groups: dental scaling, prosthodontics, endodontics, oral surgery, and periodontics.

### Covariates

We extracted the demographic information of each participant, including age, gender, socioeconomic status, urbanization, and geographic region. We also the identified the following comorbidities of BPPV within 6 months before the index date: head trauma (ICD-9-CM: 800–804, 850–854), osteoporosis (ICD-9-CM: 733.0X), migraine (ICD-9-CM: 346), hypertension (ICD-9-CM: 401–405), diabetes mellitus (ICD-9-CM: 250) hyperlipidemia (ICD-9-CM: 272.0–272.4), and ischemic or hemorrhagic stroke (ICD-9-CM: 430–434). In addition, Charlson Comorbidity Index Score (CCIS)[15] was computed to represent a range of comorbid status.

### Statistics

Data management and statistical analysis were performed using SAS 9.2 software (SAS Institute, Cary, NC). The χ2 test was used to compare the history of dental procedures, demographic data, and comorbidities between the BPPV and control groups. Odds ratios (ORs) and related 95% confidence intervals (CIs) were calculated to examine the correlations between a history of dental procedures and BPPV using multivariate logistic regression analysis after adjusting for demographic factors and comorbidities. We also use multivariate logistic regression analysis to investigate the associations between different dental procedures and BPPV. A two-sided probability value less than 0.05 was considered to be statistically significant.

## Results

Table 1 shows the baseline characteristics of the study subjects. In total, 768 patients with BPPV and 1536 age- and gender-matched controls were recruited in this study. The mean age (± SD) of the participants was 57±15 years, and 62.9% of them were female. In terms of comorbidities, CCIS was significant higher in the BPPV group (*P*<0.001); in addition, hypertension, hyperlipidemia, and migraine were significantly more prevalent in the BPPV group than in the controls (*P*<0.05). The prevalence rates of head trauma, stroke and diabetes were higher in the BPPV group than in the control group, but the differences did not reach statistical significance due to a low 6-month prevalence rate. More than half of the study population lived in un-urbanized areas, however socioeconomic status and the level of urbanization were not significantly associated with BPPV.

**Table 1:** Baseline characteristics.

Characteristics	BPPV group	Control group	*P*-value
Patient no.	768	1536	
Mean age, years (±SD)	57±15	57±15	0.365
CCIS (Mean±SD)	0.6±1.1	0.4±0.9	<0.001
Gender			NA
Male	285(37.1)	570(37.1)	
Female	483(62.9)	966(62.9)	
Comorbidities			
Hypertension	229(29.8)	322(21.0)	<0.001
Hyperlipidemia	88(11.5)	115(7.5)	0.002
Head trauma	11(1.4)	11(0.7)	0.096
Osteoporosis	13(1.7)	28(1.8)	0.824
Migraine	14(1.8)	7(0.5)	0.001
Stroke	17(2.2)	26(1.7)	0.384
Diabetes mellitus	76(9.9)	133(8.7)	0.330
Socioeconomic status			0.451
Low SES	360(46.9)	747(48.6)	
Moderate SES	261(34.0)	482(31.4)	
High SES	147(19.1)	307(20.0)	
Urbanization			0.356
Urban	248(32.3)	467(30.4)	
Un-urban	520(67.7)	1069(69.6)	
Geographic region			0.007
Northern Taiwan	499(65.0)	1083(70.5)	
Southern Taiwan	269(35.0)	453(29.5)	

BPPV = benign paroxysmal positional vertigo; CCIS = Charlson Comorbidity Index Score; SES = socioeconomic status

Table 2 shows comparisons of dental procedures between the BPPV group and control group. In the BPPV group, 9.2% of the patients had received dental procedures within 1 month before the diagnosis of BPPV. In contrast, only 5.5% of the controls had undergone dental treatment within 1 month before the index date. The rate of dental procedures was significantly higher in the BPPV group than in the control group (*P* = 0.001). When we expanded the time period from 1 month to 3 months, the rate of dental procedures was still significantly higher in the BPPV patients than in the controls (18.8% vs. 11.7%, *P*<0.001).

**Table 2:** Comparison of dental procedures between patients with and without benign paroxysmal positional vertigo.

Characteristics	BPPV group	Control group	*P*-value
Dental procedure within 1 month before the index date			0.001
Yes	71(9.2)	84(5.5)	
No	697(90.8)	1452(94.5)	
Dental procedure within 3 months before the index date			<0.001
Yes	144(18.8)	179(11.7)	
No	624(81.3)	1357(88.3)	

BPPV = benign paroxysmal positional vertigo

Table 3 presents the results of multivariate logistic regression analysis after adjusting for demographic factors and comorbidities. Compared to the subjects who did not receive dental procedures within 1 month before the index date, the adjusted OR (aOR) of BPPV was 1.77 (95% CI 1.27–2.47) for those who did undergo a dental procedure within 1 month before the index date. This association was still significant for those who underwent a dental procedure within 3 months (aOR 1.77; 95% CI 1.39–2.26). Hypertension (aOR 1.63; 95% CI 1.29–2.04), hyperlipidemia (aOR 1.46; 95% CI 1.06–1.99) and migraine (aOR 4.23; 95% CI 1.68–10.67) were independent risk factors significantly associated with BPPV.

**Table 3:** Odds ratios for benign paroxysmal positional vertigo with regards to dental procedures and comorbidities.

Variable	BPPV	
	Crude OR(95% CI)	Adjusted OR*(95% CI)
Dental procedure		
Within 1 month	1.76(1.27–2.45)	1.77(1.27–2.47)
Within 3 months	1.75(1.38–2.22)	1.77(1.39–2.26)
Comorbidities		
Hypertension	1.60(1.32–1.95)	1.63(1.29–2.04)
Hyperlipidemia	1.60(1.19–2.14)	1.46(1.06–1.99)
Head trauma	2.02(0.87–4.67)	1.87(0.79–4.44)
Osteoporosis	0.93(0.48–1.80)	0.85(0.43–1.68)
Migraine	4.06(1.63–10.09)	4.23(1.68–10.67)
Stroke	1.32(0.71–2.44)	1.07(0.56–2.05)
Diabetes mellitus	1.16(0.86–1.56)	0.90(0.65–1.25)

BPPV = benign paroxysmal positional vertigo; OR = odds ratio; CI = confidence interval

*Adjusted for age, gender, hypertension, hyperlipidemia, head trauma, osteoporosis, migraine, stroke, diabetes, socioeconomic status, urbanization and geographical region.

Table 4 lists the ORs for the risks associated with different kinds of dental procedures for BPPV diagnosed within 1 month. Three of the five procedures significantly increased the risk of BPPV, including prosthodontics (aOR 1.61; 95% CI 1.01–2.59), oral surgery (aOR 2.24; 95% CI 1.41–3.56), and periodontics (aOR 3.35; 95% CI 1.99–5.63). The other two procedures also tended to increase the risk of BPPV, but without statistical significance.

**Table 4:** Odds ratios for benign paroxysmal positional vertigo associated with different kinds of dental procedures.

Variable	1-month risk of BPPV	
	Crude OR(95% CI)	Adjusted OR*(95% CI)
Without dental procedures	1	1
Dental scaling	1.43(0.93–2.21)	1.42(0.91–2.21)
Prosthodontics	1.61(1.01–2.56)	1.61(1.01–2.59)
Endodontics	1.35(0.63–2.88)	1.36(0.63–2.93)
Oral surgery	2.15(1.36–3.40)	2.24(1.41–3.56)
Periodontics	3.36(2.01–5.61)	3.35(1.99–5.63)

BPPV = benign paroxysmal positional vertigo; OR = odds ratio; CI = confidence interval

*Adjusted for age, gender, hypertension, hyperlipidemia, head trauma, osteoporosis, migraine, stroke, diabetes, socioeconomic status, urbanization and geographical region.

## Discussion

This study demonstrates that dental procedures are a modest risk factor for BPPV, with a 1.77-fold higher odds of BPPV for those receiving dental treatment than for those without undergoing a procedure regardless of whether the diagnosis of BPPV was within 1 month or 3 months of the procedure. Although a few studies have reported on BPPV after dental therapy, most have been case reports or case series[9, 11, 13] and not systemic studies. Our study is the first population-based study to confirm a correlation between BPPV and dental procedures. In addition, most previous reports have focused on the tapping effect of osteotomes, a tool used in dental procedures[12, 14]. For example, the only previously reported control trial compared the risk of BPPV between the use of mallet and screwable osteotomes[10], whereas our results show that the risk of BPPV is increased with multiple kinds of common dental procedures such as prosthodontics, oral surgery, and periodontics.

The risk factors for BPPV can be categorized as vascular and mechanical. Among the vascular factors, migraine has been strongly associated with BPPV, with the prevalence of migraine in patients with BPPV reported to be twice that of controls[5]. Vasospasm or extravasation in the inner ear may be the underlying pathophysiology. Hypertension, diabetes and hyperlipidemia, which are causes of atherosclerosis, have also been reported to be predisposing factors for BPPV[1, 16]. Mechanical factors are also important, however. In addition to head trauma[2], which has been recognized to be a direct cause of BPPV, bed rest in a specific position and intensive body shaking have both been associated with the development of BPPV. Gyo reported that prolonged bed rest may cause loosening of otoconia which then contributes to BPPV[4]. In addition, the direction of otolith dislodgement often corresponds to the direction on which side the patient prefers to lie. In terms of vibratory impact, BPPV following mountain biking[17] or after using a whole body vibration training plate[18] has been reported. On the basis of the results of this study, we suggest that dental procedures are also a mechanical cause of BPPV, regardless of a vibratory or positional effect.

The precise pathophysiology of dental procedure-induced BPPV is unknown. One hypothesis is that the vibratory or percussive tools applied in dental therapy directly induce BPPV. Although the vibratory and percussive impacts are restricted to the oral cavity, the energy conveyed via bone may enter labyrinths and result in loosening and dislodgement of otoliths. Another hypothesis suggests that repeated sitting up and lying down during dental treatment, sometimes with a head position below the horizon, may displace otoliths thereby inducing BPPV.

If the mechanical effects of dental procedures induce BPPV immediately, the date of a diagnosis of BPPV should be close to the date of dental therapy with an interval of less than 1 month. However, when we expanded the time period from 1 month to 3 months, the OR of BPPV did not decrease. Therefore, we suggest that dental procedures sometimes just initially loosen otoconia, and then dislodgement of otoliths may be delayed for days, weeks or even months.

There are several limitations to this study. First, this study is a retrospective analysis using data from the LHID, so we cannot ensure the accuracy of the diagnoses of BPPV. In order to eliminate the effect of this natural limitation of a database, we tried to reduce the diagnostic uncertainty as far as possible by excluding the patients whose BPPV diagnosis was only recorded in one or two out-patient department follow-up visits, and excluded the patients with multiple diagnoses of vestibular disorders. Inevitably these exclusion criteria made us miss the patients who were only treated in one or two sessions and the patients who actually had multiple vestibular disorders. Second, a few dental procedures which are not covered by the National Health Insurance program such as dental implantation and orthodontics were not included in our analysis. Third, according to the LHID, the index date of the BPPV group was the first date of a diagnosis of BPPV during the inclusion period for each patient. Therefore, we cannot exclude the possibility that some patients already had BPPV before undergoing dental therapy. Changing position during dental therapy may just highlight the symptoms of BPPV, prompting the patients to seek medical care. However, we assumed that the likelihood of this was low, because the symptoms of BPPV are readily detected by the patients themselves during sleep and during daily activities or exercise. In addition, if most cases of BPPV were pre-existing before dental treatment, the 1-month OR for BPPV would be much higher than the 3-month OR, which was not the case.

## Conclusions

This study demonstrates a correlation between dental procedures and BPPV. The finding not only clarifies the mechanical pathophysiology of BPPV, but also provides important clinical clues. We suggest that specialists who treat BPPV should ask about dental procedures when taking the patient’s history, and emphasize the importance of dental care to avoid frequent dental procedures for high-risk patients. In addition, dentists should recognize that BPPV is one of the complications of dental treatment, be able to identify it, and refer these patients to suitable specialists.
